# Supplementary material for: Co-evolution networks of HIV/HCV are modular with direct association to structure and function
Source: PLoS Comput Biol. 2018 Sep 7;14(9):e1006409. doi: 10.1371/journal.pcbi.1006409 (PMC6145588; doi:10.1371/journal.pcbi.1006409)
Supplement: S1 Text — (PDF) [file pcbi.1006409.s001.pdf]

Supplementary Information

Co-evolution networks of HIV/HCV are modular with direct association to structure and function

Ahmed Abdul Quadeer[1], David Morales-Jimenez[2], Matthew R. McKay[1,3*],

1 Department of Electronic and Computer Engineering, The Hong Kong University of Science and Technology, Hong Kong.
2 Institute of Electronics, Communications and Information Technology, Queen's University Belfast, Belfast, UK.
3 Department of Chemical and Biological Engineering, The Hong Kong University of Science and Technology, Hong Kong.

*Corresponding author: m.mckay@ust.hk

S1 Text. Details of the PCA-based inference method [1]

Similar to RoCA, the PCA-based method [1] is based on the Pearson correlation matrix, but it differs markedly from RoCA in the eigenvector estimation approach: PCA naively performs a classical spectral decomposition (i.e., it directly uses $\mathbf{q}_k$ (see Eq (2) in Materials and Methods) as the eigenvector estimates), while RoCA involves a state-of-the-art sparse estimation method, as detailed in the Materials and Methods section. PCA sectors are formed by thresholding the sample eigenvectors $\mathbf{q}_k$ (using a small threshold $\epsilon = 0.005$) to disregard spurious coordinates [1]. Note that, due to the prominent statistical noise in $\mathbf{q}_k$ (evident in Fig 4B and S4 Fig), such thresholding procedure of PCA results in very large sectors as compared with RoCA, where the sparse eigenvector estimates $\mathbf{p}_k$ are used instead (S3 Fig). Thus in [1], the sectors obtained from this initial thresholding are further post-processed to (i) remove all residues with no significant intra-sector correlations, and (ii) *enforce the sectors to be non-overlapping.* These constrained post-processing steps are not present in RoCA and, as such, RoCA sectors may comprise a few overlapping residues (gray circles in Fig 2A and S1 Fig). Implementation details of PCA can be found in [1].

References

1. Quadeer AA, Louie RHY, Shekhar K, Chakraborty AK, Hsing IM, McKay MR. Statistical linkage analysis of substitutions in patient-derived sequences of genotype 1a hepatitis C virus nonstructural protein 3 exposes targets for immunogen design. J Virol. 2014;88(13):7628–44. doi:10.1128/JVI.03812-13.
