# Supplementary material for: Co-evolution networks of HIV/HCV are modular with direct association to structure and function
Source: PLoS Comput Biol. 2018 Sep 7;14(9):e1006409. doi: 10.1371/journal.pcbi.1006409 (PMC6145588; doi:10.1371/journal.pcbi.1006409)
Supplement: S2 Text — (PDF) [file pcbi.1006409.s002.pdf]

Supplementary Information

Co-evolution networks of HIV/HCV are modular with direct association to structure and function

Ahmed Abdul Quadeer[1], David Morales-Jimenez[2], Matthew R. McKay[1,3*],

1 Department of Electronic and Computer Engineering, The Hong Kong University of Science and Technology, Hong Kong.
2 Institute of Electronics, Communications and Information Technology, Queen's University Belfast, Belfast, UK.
3 Department of Chemical and Biological Engineering, The Hong Kong University of Science and Technology, Hong Kong.

*Corresponding author: m.mckay@ust.hk

S2 Text. Simulation study: Statistical robustness of RoCA

To more precisely test the inference accuracy of RoCA, we developed a ground-truth simulation model and studied the performance under different sampling conditions (i.e., for different ratios of the number of sequences N to the protein length M). We also studied the accuracy of the PCA-based sectoring approach [1] (which we referred to as simply "PCA") that, as discussed above, fundamentally differs from RoCA. The simulation model generates correlated binary samples with specified *units*, with parameters set close to those observed for the HIV Gag data set. Note that we use the term "unit" to specify each *ground-truth* group of correlated residues, in order to clearly differentiate from a "sector", which corresponds to an *inferred* group of correlated residues. For better readability, the main results are presented first, while a detailed description of the model and the utilized performance metrics is given subsequently.

Simulation results: RoCA is robust to limited sampling, PCA is very sensitive

Our results (S3 Fig) demonstrate that the RoCA sectors accurately infer the individual units, showing a mean true positive rate (TPR) close to 100% and a mean false discovery rate (FDR) below 5% even under stringent sampling (i.e., when N/M is low). The accuracy of the PCA sectors, on the other hand, deteriorates substantially when faced with limited data. Specifically, for low N/M, the median value of the mean TPR is close to 0.6 (S3 Fig), indicating that in a typical simulation run, only 60% of the unit residues are correctly inferred on average. Moreover, the median value of the mean FDR in this case is approximately 0.3 (S3 Fig), demonstrating that around 30% of the residues in the PCA sectors are incorrect on average.

While the TPR and FDR are informative measures of the inference of individual units, they do not reflect the ability to collectively infer all *distinct* units, i.e., an inferred sector with relatively high TPR and low FDR may still merge residues from different units. To more precisely test this feature, we introduce the "maximum percentage mismatch" $\mathrm{PM}_{\max}$ (see Eq (6) below), with a smaller value of $\mathrm{PM}_{\max}$ implying better inference. In terms of this measure, our results (S3 Fig) demonstrate that RoCA has excellent ability to classify distinct units, achieving perfect unit discrimination under all scenarios tested for most simulation runs (the median value is exactly 0 for each value of N/M). PCA, on the other hand, tends to form larger sectors which represent a mixture of residues from multiple units, with this effect being more pronounced under more stringent sampling (S3 Fig). Specifically, the median value of $\mathrm{PM}_{\max}$ for PCA is approximately 90% for low N/M, indicating that nearly all residues of at least one unit are merged with those of another unit in one of the inferred sectors.

In summary, the above tests demonstrate the superior robustness and discriminatory power of the RoCA sectors compared with those produced by PCA.

Detailed description of the simulation model

We defined a simple r-unit correlation matrix construction of dimension $M \times M$,

$$\mathbf{\Gamma} = (\mathbf{I}_M - \mathbf{Z}) + \underbrace{\sum_{k=1}^{r} \ell_k \mathbf{u}_k \mathbf{u}_k^{\mathsf{T}}}_{r \text{ units}} + \underbrace{\ell_0 \mathbf{u}_0 \mathbf{u}_0^{\mathsf{T}}}_{\text{Phylogeny}} , \tag{1}$$

where $\mathbf{Z}$ is a diagonal matrix with elements $Z_{ii} = \sum_{k=0}^{r} \ell_k \mathbf{u}_k(i)^2$, $\ell_1 \geq \ldots \geq \ell_r > 0$ are scalars governing the strength of the intra-unit correlations for units $1, \ldots, r$, respectively, and $\mathbf{u}_1, \ldots, \mathbf{u}_r$ are orthonormal vectors whose support (non-zero entries) define these units, i.e., the kth unit is defined as the set $S_k = \{i \in \{1, \ldots, M\} : \mathbf{u}_k(i) \neq 0\}$. Only non-overlapping supports were considered to model distinct units, i.e., $S_i \cap S_j = \emptyset$ for every $i \neq j$, and the supports were chosen to be rather small, resulting in sparse units and a large proportion of uncorrelated residues. The phylogenetic effect was modeled by ℓ_0, with $\ell_0 > \ell_1$, and $\mathbf{u}_0 = \frac{1}{\sqrt{M}}[1\ 1\ \ldots\ 1]^{\mathsf{T}}$. The parameters $\{M, r, \ell_k, \mathbf{u}_k\}$ were set to yield similar characteristics to those observed for the HIV Gag data set.

Synthetic binary sequences of length M were generated according to the specified correlation $\mathbf{\Gamma}$ and with mutation probabilities f_i, $i = 1, \ldots, M$, by applying the Emrich-Piedmonte (EP) method [2]. Note however that, due to the natural constraints of binary data, not every choice of $\{\mathbf{\Gamma}, f_i\}$ is feasible. Specifically, the correlation coefficient Γ_{ij} must satisfy [2]

$$\max\left\{-\left(\frac{f_i f_j}{g_i g_j}\right)^{1/2}, -\left(\frac{g_i g_j}{f_i f_j}\right)^{1/2}\right\} \leq \Gamma_{ij} \leq \min\left\{\left(\frac{f_i g_j}{f_j g_i}\right)^{1/2}, \left(\frac{f_j g_i}{f_i g_j}\right)^{1/2}\right\}, \tag{2}$$

where $g_i = 1 - f_i$. Consistent with the viral data sets analyzed in this work, the mutation probabilities f_i were set to rather small values; however, in order to satisfy the feasibility constraints (Eq (2)) and to avoid computational issues, these probabilities needed to be slightly higher than the typical mutation frequencies observed in the HIV Gag data set. In particular, each f_i ($i = 1, \ldots, M$) was randomly chosen according to a uniform distribution between 0.1 and 0.2.

In order to assess the inference accuracy, we first generated a large number $\bar{N}$ of synthetic binary sequences. Then, for each choice of N/M, the following Monte Carlo simulation procedure was repeated for 500 iterations:

1. A binary MSA was formed by drawing N samples (sequences) without replacement from the $\overline{N}$ previously generated ones.

2. Sectors were inferred using the method under study (RoCA or PCA).

3. The obtained sectors may not correspond to the true units (Eq (1)). For example, sector 1 may be predicting unit 2 instead of 1. We rearranged the sectors in order for them to be best aligned with the true units. To that end, all $r!$ sectors permutations were tested and the best permutation, κ^*, was chosen according to

$$\kappa^* = \arg\max_{\kappa} \left(\frac{1}{r} \sum_{i=1}^{r} \frac{\mathrm{TP}_i^{\kappa} - \mathrm{TN}_i^{\kappa}}{M}\right), \quad \kappa = 1, 2, ..., r! \tag{3}$$

where, TP_i^{κ} and TN_i^{κ} are respectively the number of true positives and true negatives associated with sector i of permutation κ. These quantify the accuracy of the prediction [3] for a given sector-unit pair and are defined as

$$\mathrm{TP}_i^{\kappa} = \#(S_i \cap \hat{S}_i^{\kappa}) \quad \text{and} \quad \mathrm{TN}_i^{\kappa} = \#(S_i^c \cap \hat{S}_i^{c^{\kappa}}), \tag{4}$$

where S_i is the set of residues in the ith unit, $\hat{S}_i^{\kappa}$ is the set of residues in the ith sector of permutation κ, x^c is the complement of set x, and $\#(x)$ denotes the cardinality of set x.

4. With sectors and units aligned in accordance with κ^*, the inference accuracy for each sector-unit pair was characterized using two metrics: the TPR, representing the proportion of the unit residues that are correctly included in the sector, and the FDR, representing the proportion of the sector residues that are not in the specified units. Specifically, for each sector i, these metrics are defined as

$$\mathrm{TPR}_i = \frac{\#(S_i \cap \hat{S}_i^{\kappa^*})}{\#(S_i)} \quad \text{and} \quad \mathrm{FDR}_i = \frac{\#(S_i^c \cap \hat{S}_i^{\kappa^*})}{\#(\hat{S}_i^{\kappa^*})}. \tag{5}$$

5. To further characterize the one-to-one correspondence between sectors and their corresponding units (as indicated earlier, this is not implied by a high TPR and low FDR), we introduced the "maximum percentage mismatch" performance metric,

$$\mathrm{PM}_{\max} = \max\left\{ \frac{\#\left(S_i \cap \hat{S}_j^{\kappa^*}\right)}{\#\left(S_i\right)} \times 100, \quad i, j = 1, 2, \cdots, r \ \text{ and } j \neq i \right\}. \tag{6}$$

This quantifies the decoupling power of a given method; that is, the ability to discriminate distinct units based on the obtained sectors. A low value of $\mathrm{PM}_{\max}$ (close to 0) signifies the ideal one-to-one correspondence between sectors and units, whereas a high value implies that a large proportion of a unit is included in the wrong sector. The latter implies that multiple units are merged into a single sector, and hence they are not distinctly inferred.

6. The mean TPR ($\frac{1}{r}\sum_{i=1}^{r} \mathrm{TPR}_i$), mean FDR ($\frac{1}{r}\sum_{i=1}^{r} \mathrm{FDR}_i$), and the $\mathrm{PM}_{\max}$ were recorded.

References

1. Quadeer AA, Louie RHY, Shekhar K, Chakraborty AK, Hsing IM, McKay MR. Statistical linkage analysis of substitutions in patient-derived sequences of genotype 1a hepatitis C virus nonstructural protein 3 exposes targets for immunogen design. J Virol. 2014;88(13):7628–44. doi:10.1128/JVI.03812-13.

2. Emrich LJ, Piedmonte MR. A method for generating high-dimensional multivariate binary variates. Am Stat. 1991;45(4):302–304. doi:10.1080/00031305.1991.10475828.

3. Metz CE. Basic principles of ROC analysis. Semin Nucl Med. 1978;8(4):283–298. doi:http://dx.doi.org/10.1016/S0001-2998(78)80014-2.
