# Supplementary material for: Co-evolution networks of HIV/HCV are modular with direct association to structure and function
Source: PLoS Comput Biol. 2018 Sep 7;14(9):e1006409. doi: 10.1371/journal.pcbi.1006409 (PMC6145588; doi:10.1371/journal.pcbi.1006409)
Supplement: S3 Text — (PDF) [file pcbi.1006409.s003.pdf]

Supplementary Information

Co-evolution networks of HIV/HCV are modular with direct association to structure and function

Ahmed Abdul Quadeer[1], David Morales-Jimenez[2], Matthew R. McKay[1,3*],

1 Department of Electronic and Computer Engineering, The Hong Kong University of Science and Technology, Hong Kong.
2 Institute of Electronics, Communications and Information Technology, Queen's University Belfast, Belfast, UK.
3 Department of Chemical and Biological Engineering, The Hong Kong University of Science and Technology, Hong Kong.

*Corresponding author: m.mckay@ust.hk

S3 Text. Analysis of the SCA method

Statistical coupling analysis (SCA) is a benchmark co-evolution method which has been employed to infer sectors of co-evolving residues with strong biochemical association in various protein families [1–4]. For the studied HIV and HCV proteins however, SCA produced sectors with little or no significant association to known biochemical domains (S5 Fig). Here we provide further details of this method and analyze fundamental differences with respect to the RoCA approach.

SCA is based on a conservation-weighted covariance matrix, $\mathbf{\Omega}^{\mathrm{SCA}}$, directly computed from the binary MSA. In contrast, RoCA is based on the Pearson correlation matrix, computed from the phylogeny-filtered MSA (Materials and Methods); note however that SCA does also filter the phylogenetic effect, in an alternative but similar way, by discarding the leading eigenvector prior to forming sectors [1]. In the subsequent description, to simplify the discussion, the covariance and correlation coefficients refer to those computed from the (unfiltered) binary MSA and we use different notation to avoid potential confusion with the corresponding quantities defined in Materials and Methods.

To specify the SCA matrix, first denote f_i the mutation frequency at residue i, and f_{ij} the pairwise mutation frequency at residues i and j. Also, let a denote the consensus amino acid at residue i, having frequency $g_i^a = 1 - f_i$, which we refer to as the "conservation" of residue i. With these definitions, the entries of the SCA matrix $\mathbf{\Omega}^{\mathrm{SCA}}$ are given by

$$\Omega_{ij}^{\mathrm{SCA}} = \phi_i \phi_j \left|\Omega_{ij}\right|, \quad i, j = 1, 2, ..., M, \tag{1}$$

where $\Omega_{ij} = f_{ij} - f_i f_j$ represents the mutational covariance between residues i and j, while ϕ_i is a residue-specific weight defined as $\phi_i = \ln \frac{g_i^a(1-q^a)}{q^a(1-g_i^a)}$. This weight involves the consensus frequency g_i^a as well as the quantity q^a, representing the background frequency of amino acid a observed across all proteins in nature. Based on $\mathbf{\Omega}^{\mathrm{SCA}}$, SCA employs spectral analysis with the aim of identifying correlated groups of residues while simultaneously emphasizing those residues which are most conserved. The weight ϕ_i varies with the frequency of the consensus amino acid at residue i, calibrated against how frequent this amino acid is in nature. For our cases of interest, we always have $g_i^a > q^a$, and ϕ_i increases non-linearly with increased conservation (i.e., as g_i^a increases).

It is instructive to express the entries of the SCA matrix in terms of the correlation coefficients as

$$\Omega_{ij}^{\mathrm{SCA}} = \underbrace{\phi_i \sigma_i}_{w_i} \underbrace{\phi_j \sigma_j}_{w_j} \left|R_{ij}\right|, \quad i, j = 1, 2, ..., M, \tag{2}$$

where $\sigma_i = \sqrt{f_i(1-f_i)}$ is the mutational standard deviation for residue i and $R_{ij} = \frac{\Omega_{ij}}{\sigma_i \sigma_j}$ is the correlation coefficient between residues i and j. In this representation, w_i is seen as the weight (relative to correlation) associated with residue i in the SCA matrix. By definition, the correlation coefficients R_{ij} are independent of conservation, hence these weights clearly quantify the role of conservation in the SCA matrix construction. To shed light into such effect, consider the diagonal entries $\Omega_{ii}^{\text{SCA}} = w_i^2$ (recall that $R_{ii} = 1$). These are plotted in Fig A as a function of conservation g_i^a, with the background frequency q^a set to the average value taken over all amino acids. (Note that q^a is quite small for all amino acids, ranging from 0.01 to 0.09 and, consequently, the trends observed in Fig A remain the same for any specific choice of amino acid a.) As seen from the figure, for the range of conservation values $g_i^a \sim$[0.1, 0.75], which embraces those values generally observed for protein families (on which SCA was applied), the SCA weights monotonically boost the more conserved residues, as expected. However, for the much higher conservation levels observed in each of the viral proteins (i.e., $g_i^a > 0.8$), the behavior is dramatically different—the SCA weights in this case *depress* the most conserved residues. In this sense, for the highly conserved viral proteins, the SCA matrix effectively behaves in a similar manner to the classical covariance matrix, assigning higher weights to those residues with greater variability. Consistent with these observations, the few SCA sectors that were found to associate to a biochemical domain corresponded to those with the lowest mean conservation (Fig A). These results suggest that the SCA matrix, at least in view of its original design objective of emphasizing mutational conservation, is well suited for the co-evolutionary analysis of certain protein families, but not for the HIV and HCV proteins under study.

SCA Implementation

For implementing SCA, we used the code provided in [1]. Note that a new implementation is also available at `http://systems.swmed.edu/rr_lab/` that involves forming sectors using the independent components, obtained from independent component analysis. However, results obtained with both implementations were nearly indistinguishable.

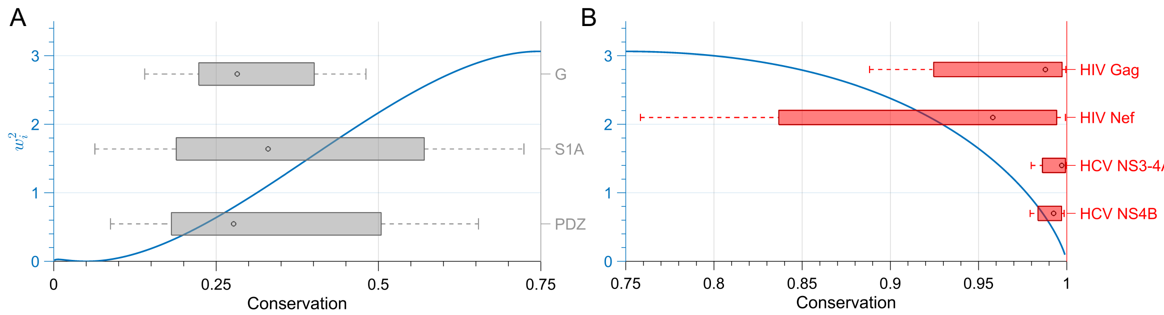

Fig A. Weighting of the protein residues in SCA with respect to conservation. The left y-axis (blue) plots the trend followed by the diagonal entries of the SCA matrix, w_i^2, as a function of conservation. The right y-axis plots the conservation of (A) three protein families that were analyzed using the SCA method (G protein-coupled receptors [5], S1A serine proteases [1] and PDZ domain family [3,6]) (gray) and (B) the four internal viral proteins studied in this work (red). The black circle indicates the median, the edges of the box represent the first and third quartiles, and whiskers extend to span a 1.5 inter-quartile range from the edges.

References

1. Halabi N, Rivoire O, Leibler S, Ranganathan R. Protein sectors: Evolutionary units of three-dimensional structure. Cell. 2009;138(4):774–786. doi:10.1016/j.cell.2009.07.038.

2. Rivoire O, Reynolds KA, Ranganathan R. Evolution-based functional decomposition of proteins. PLoS Comput Biol. 2016;12(6):e1004817. doi:10.1101/022525.

3. McLaughlin Jr RN, Poelwijk FJ, Raman A, Gosal WS, Ranganathan R. The spatial architecture of protein function and adaptation. Nature. 2012;491:138–142. doi:10.1038/nature11500.

4. Smock RG, Rivoire O, Russ WP, Swain JF, Leibler S, Ranganathan R, et al. An interdomain sector mediating allostery in Hsp70 molecular chaperones. Mol Sys Biol. 2010;6(414):1–10. doi:10.1038/msb.2010.65.

5. Süel GM, Lockless SW, Wall MA, Ranganathan R. Evolutionarily conserved networks of residues mediate allosteric communication in proteins. Nat Struct Mol Biol. 2003;10(1):59–69. doi:10.1038/nsb881.

6. Lockless SW, Ranganathan R. Evolutionarily conserved pathways of energetic connectivity in protein families. BMC Bioinformatics. 1999;15(5438):295–299. doi:10.1186/1471-2105-15-6.
