# Supplementary material for: Co-evolution networks of HIV/HCV are modular with direct association to structure and function
Source: PLoS Comput Biol. 2018 Sep 7;14(9):e1006409. doi: 10.1371/journal.pcbi.1006409 (PMC6145588; doi:10.1371/journal.pcbi.1006409)
Supplement: S1 Fig — The first row of each panel shows the biplots of the estimated sparse PCs that are used to form RoCA sectors. The sector residues are represented by circles according to the specified color scheme, while overlapping residues (belonging to more than one sector) and non-sector (independent) residues are represented as gray and white circles, respectively. The heat map of the cleaned correlation matrix, with rows and columns ordered according to the residues in the RoCA sectors, shows that the sectors are notably sparse and uncorrelated to each other. The second row of each panel plots the location of RoCA sector residues in the primary structure. The sector residues are colored according to the specifications in Fig 3A. The third row of each panel shows the statistical independence of the RoCA sectors, quantified using the normalized entropy deviation metric. (PDF) [file pcbi.1006409.s004.pdf]

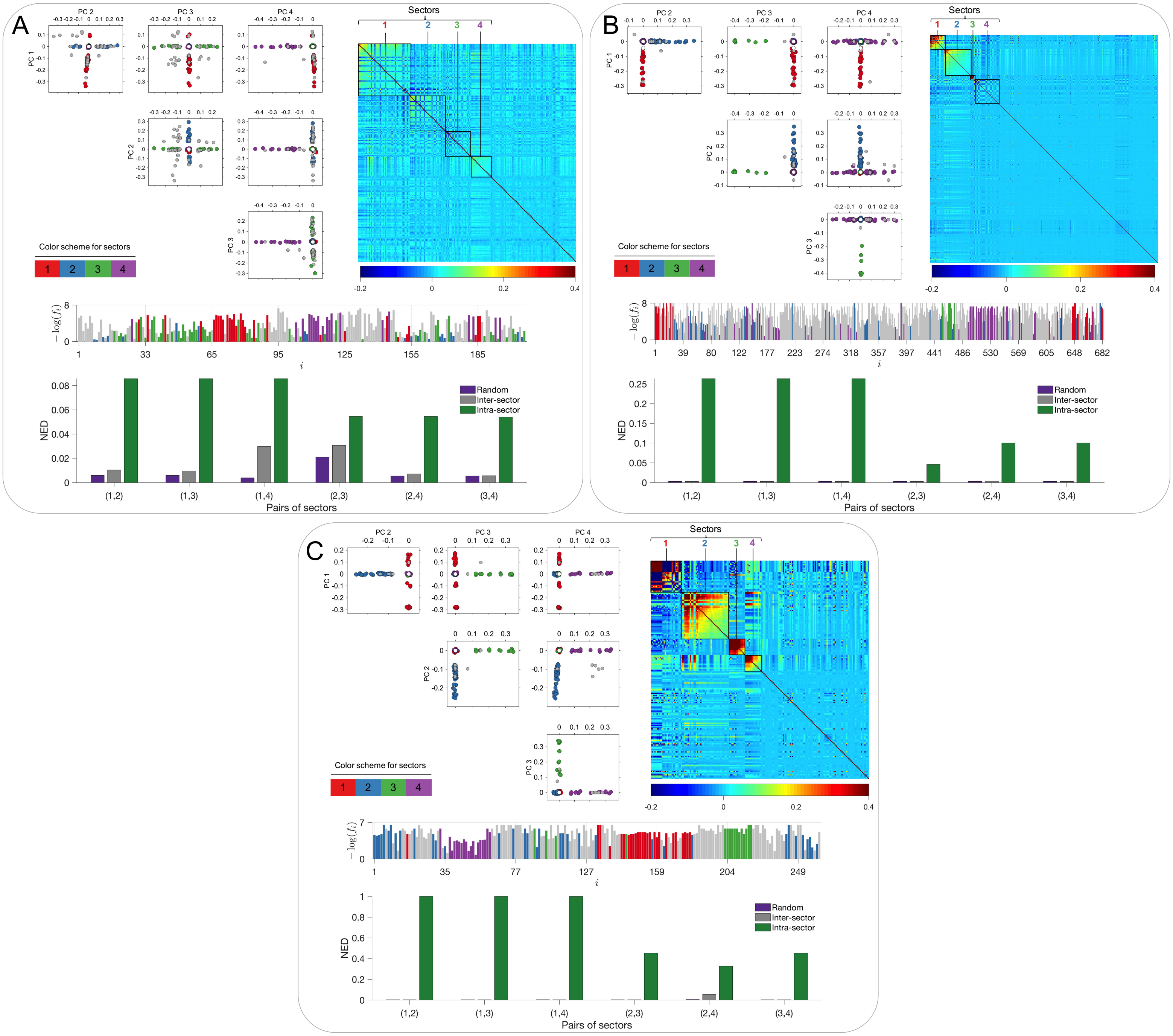
A
B
C
Sectors
Color scheme for sectors
PC 1
PC 2
PC 3
PC 4
$-\log(f_i)$
i
NED
Random
Inter-sector
Intra-sector
(1,2)
(1,3)
(1,4)
(2,3)
(2,4)
(3,4)
Pairs of sectors

Fig S1 ***(preceding page)*****. Co-evolutionary sectors revealed by RoCA for (A) HIV Nef, (B) HCV NS3-4A, and (C) HCV NS4B proteins.** The first row of each panel shows the biplots of the estimated sparse PCs that are used to form RoCA sectors. The sector residues are represented by circles according to the specified color scheme, while overlapping residues (belonging to more than one sector) and non-sector (independent) residues are represented as gray and white circles, respectively. The heat map of the cleaned correlation matrix, with rows and columns ordered according to the residues in the RoCA sectors, shows that the sectors are notably sparse and uncorrelated to each other. The second row of each panel plots the location of RoCA sector residues in the primary structure. The sector residues are colored according to the specifications in Fig 2A. The third row of each panel shows the statistical independence of the RoCA sectors, quantified using the normalized entropy deviation metric.
