# Supplementary material for: Co-evolution networks of HIV/HCV are modular with direct association to structure and function
Source: PLoS Comput Biol. 2018 Sep 7;14(9):e1006409. doi: 10.1371/journal.pcbi.1006409 (PMC6145588; doi:10.1371/journal.pcbi.1006409)
Supplement: S2 Fig — The protein chains in all structures are shown in gray. The structurally important residues (i.e., interface residues) present in a sector are shown as red spheres and the remaining residues of the interface are shown as blue spheres. The first, second, and third column show the interface residues obtained with d = 7, 8, and 9Å, respectively. Crystal structure of (A) the p24 hexamer (PDB ID 3GV2), (B) the p24 pentamer (PDB ID 3P05), and (C) the p24 inter-hexamer interface (PDB ID 2KOD). All the associated results in the main text were shown for d = 7Å. The association of sector 3 and sector 6 to the corresponding interfaces remains statistically significant for different values of d. (PDF) [file pcbi.1006409.s005.pdf]

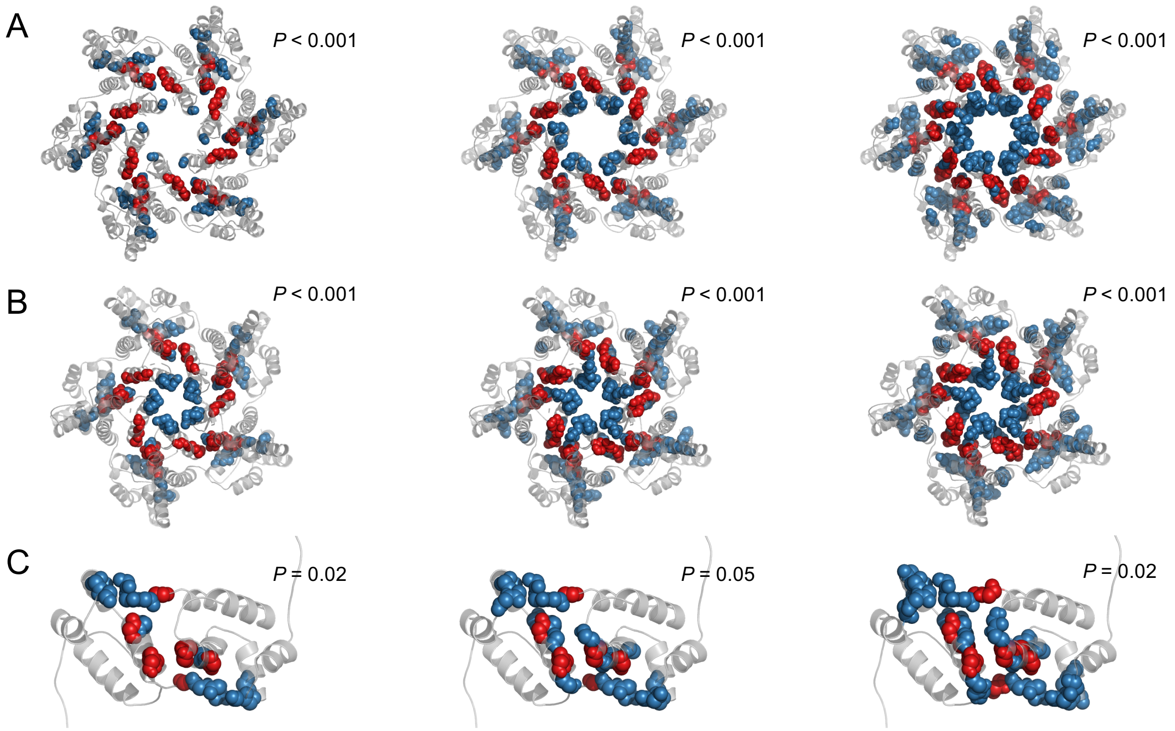

Fig S2. Robustness of the association of HIV Gag sectors 3 and 6 to the respective structural (interface) domains for different values of the contact-defining distance d between the alpha-carbon atoms. The protein chains in all structures are shown in gray. The structurally important residues (i.e., interface residues) present in a sector are shown as red spheres and the remaining residues of the interface are shown as blue spheres. The first, second, and third column show the interface residues obtained with $d = 7$, 8, and 9Å, respectively. Crystal structure of (A) the p24 hexamer (PDB ID 3GV2), (B) the p24 pentamer (PDB ID 3P05), and (C) the p24 inter-hexamer interface (PDB ID 2KOD). All the associated results in the main text were shown for $d = 7$Å. The association of sector 3 and sector 6 to the corresponding interfaces remains statistically significant for different values of d.
