# Supplementary material for: Co-evolution networks of HIV/HCV are modular with direct association to structure and function
Source: PLoS Comput Biol. 2018 Sep 7;14(9):e1006409. doi: 10.1371/journal.pcbi.1006409 (PMC6145588; doi:10.1371/journal.pcbi.1006409)
Supplement: S3 Fig — (A) Comparison of sector sizes obtained using PCA and RoCA for the studied viral proteins. The y-axis shows the number of residues present in each sector. PCA sectors were inferred by applying the method proposed in [12] on the available sequence data of each viral protein. (B) Comparison of the robustness of RoCA and PCA [12] methods to finite sampling using binary synthetic data. The parameters used in the simulation (see S2 Text for details) were M = 500 residues and r = 5 non-overlapping units Si of size 12%, 10%, 8%, 6%, and 4% of M, respectively for i = 1, …, 5. The corresponding ℓi were set to equally spaced values between ℓ1 = 6 and ℓ5 = 4, and to model the phylogenetic effect, we set ℓ0 = 8. To test the finite-sampling effect, results are presented for varying number of samples N = 1000, 2000, and 4000 corresponding to NM = 2, 4, and 8, respectively. The sectors inferred using RoCA and PCA [12] are compared using mean TPR, mean FDR, and the maximum percentage mismatch PMmax. In each box plot, the black circle indicates the median, the edges of the box represent the first and third quartiles, and whiskers extend to span a 1.5 inter-quartile range from the edges. (PDF) [file pcbi.1006409.s006.pdf]

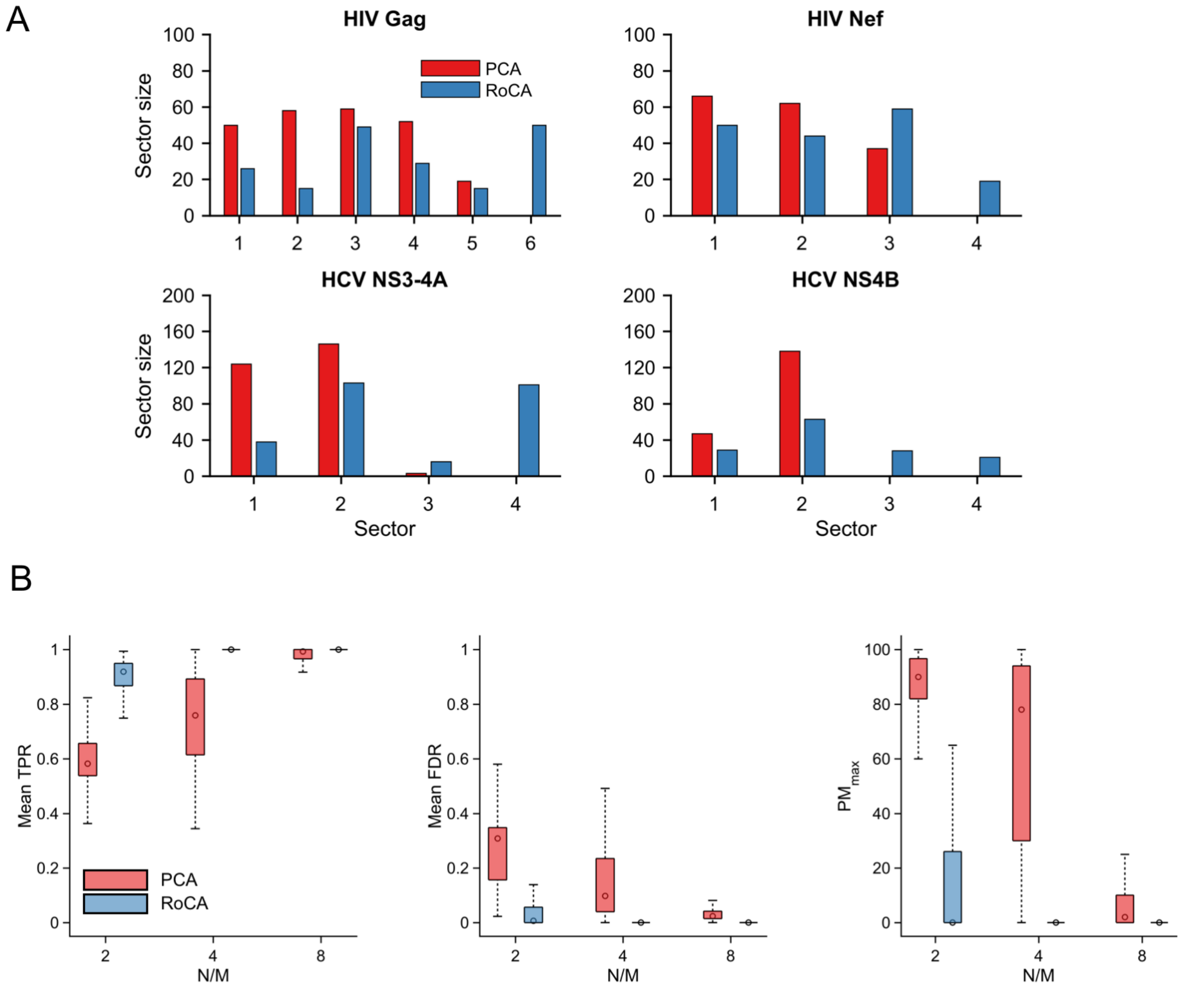

Fig S3. Comparison of the sectors inferred using the PCA-based method [1] and the proposed RoCA method. (A) Comparison of sector sizes obtained using PCA and RoCA for the studied viral proteins. PCA sectors were inferred by applying the method proposed in [1] on the available sequence data of each viral protein. The y-axis shows the number of residues present in each sector. (B) Comparison of the robustness of RoCA and PCA [1] methods to finite sampling using binary synthetic data. The parameters used in the simulation were $M = 500$ residues and $r = 5$ non-overlapping units S_i of size 12%, 10%, 8%, 6%, and 4% of M, respectively for $i = 1, \ldots, 5$. The corresponding ℓ_i were set to equally spaced values between $\ell_1 = 6$ and $\ell_5 = 4$, and to model the phylogenetic effect, we set $\ell_0 = 8$. To test the finite-sampling effect, results are presented for varying number of samples $N =$ 1000, 2000, and 4000 corresponding to $\frac{N}{M} = 2$, 4, and 8, respectively. The sectors inferred using RoCA and PCA [1] are compared using mean TPR, mean FDR, and the maximum percentage mismatch PM_{max}. In each box plot, the black circle indicates the median, the edges of the box represent the first and third quartiles, and whiskers extend to span a 1.5 inter-quartile range from the edges.

References

1. Quadeer AA, Louie RHY, Shekhar K, Chakraborty AK, Hsing IM, McKay MR. Statistical linkage analysis of substitutions in patient-derived sequences of genotype 1a hepatitis C virus nonstructural protein 3 exposes targets for immunogen design. J Virol. 2014;88(13):7628–44. doi:10.1128/JVI.03812-13.
