# Supplementary material for: Co-evolution networks of HIV/HCV are modular with direct association to structure and function
Source: PLoS Comput Biol. 2018 Sep 7;14(9):e1006409. doi: 10.1371/journal.pcbi.1006409 (PMC6145588; doi:10.1371/journal.pcbi.1006409)
Supplement: S4 Fig — The first column displays the bar plots showing the merging of multiple RoCA sectors in the sectors revealed by the method in [12]. The vertical axis of each plot shows the percentage of residues within the different RoCA sectors that fall into the prescribed sector. The second column shows the biplots of the PCs which are post-processed to form sectors in [12]. The sector residues are represented by circles according to the specified color scheme, while independent (non-sector) residues are represented as white circles. (PDF) [file pcbi.1006409.s007.pdf]

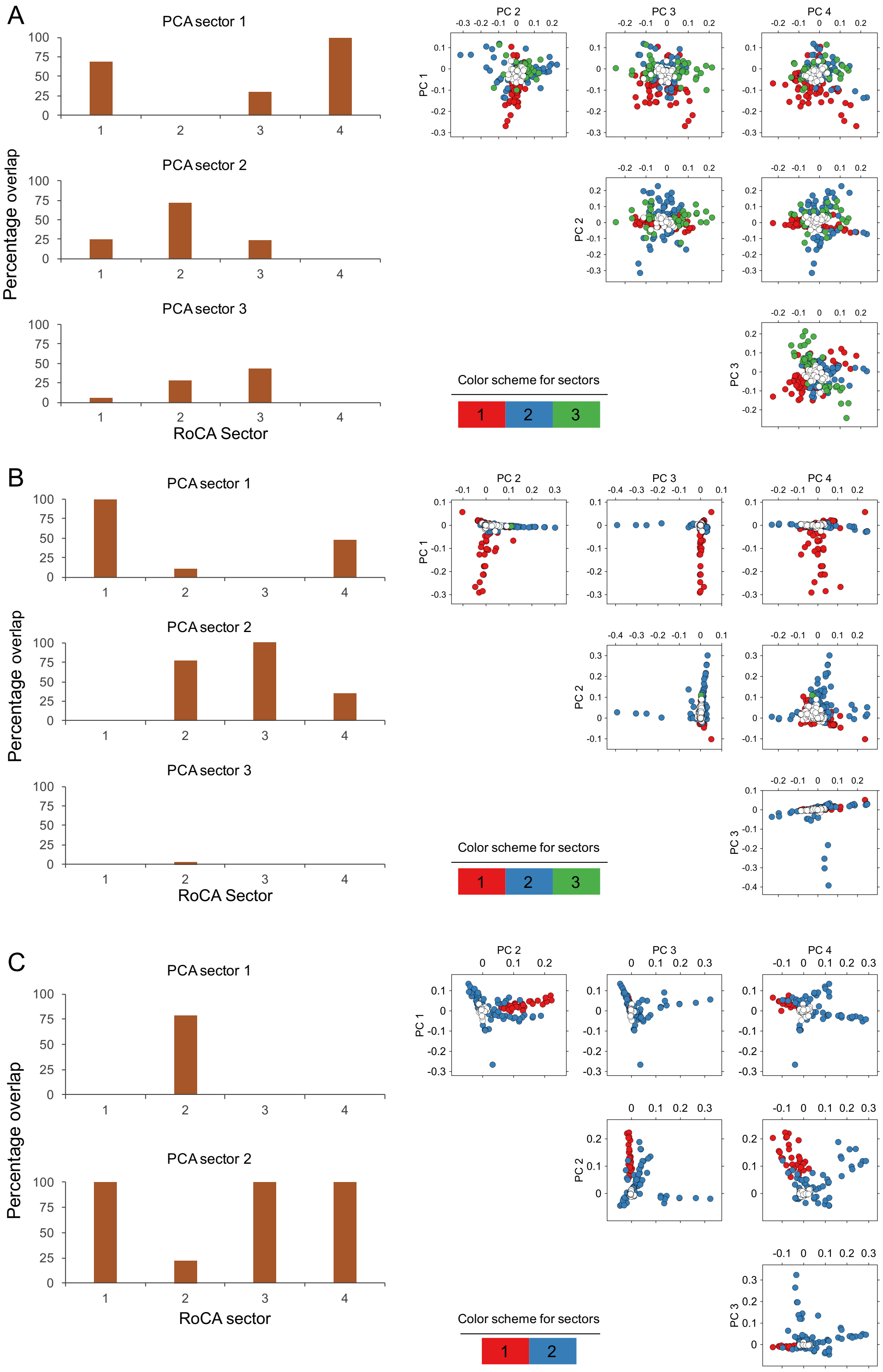
A
PCA sector 1
PCA sector 2
PCA sector 3
Percentage overlap
RoCA Sector
PC 1
PC 2
PC 3
PC 4
Color scheme for sectors
1
2
3
B
PCA sector 1
PCA sector 2
PCA sector 3
Percentage overlap
RoCA Sector
PC 1
PC 2
PC 3
PC 4
Color scheme for sectors
1
2
3
C
PCA sector 1
PCA sector 2
Percentage overlap
RoCA sector
PC 1
PC 2
PC 3
PC 4
Color scheme for sectors
1
2

Fig S4 ***(preceding page).*** **Sectors revealed by applying the PCA-based method [1] on the available sequence data of (A) HIV Nef, (B) HCV NS3-4A, and (C) HCV NS4B proteins.** The first column displays the bar plots showing the merging of multiple RoCA sectors in the sectors revealed by the PCA method [1]. The vertical axis of each plot shows the percentage of residues within the different RoCA sectors that fall into the prescribed sector. The second column shows the biplots of the PCs which are post-processed to form sectors in the PCA method [1]. The sector residues are represented by circles according to the specified color scheme, while independent (non-sector) residues are represented as white circles.

References

1. Quadeer AA, Louie RHY, Shekhar K, Chakraborty AK, Hsing IM, McKay MR. Statistical linkage analysis of substitutions in patient-derived sequences of genotype 1a hepatitis C virus nonstructural protein 3 exposes targets for immunogen design. J Virol. 2014;88(13):7628–44. doi:10.1128/JVI.03812-13.
