# Supplementary material for: Co-evolution networks of HIV/HCV are modular with direct association to structure and function
Source: PLoS Comput Biol. 2018 Sep 7;14(9):e1006409. doi: 10.1371/journal.pcbi.1006409 (PMC6145588; doi:10.1371/journal.pcbi.1006409)
Supplement: S5 Fig — (A) Individual associations of sectors produced by the SCA method proposed in [14] with the biochemical domains of the studied viral proteins; compare with Figs 4 and 5C. The sectors are colored according to the scheme in Fig 3A. Only the sectors having statistically significant association with any biochemical domain are presented. The P-values associated with non-significant associations (P > 0.05) are displayed inside the black circle while the biochemical domains having no association with any inferred sector (P = 1) are shown in bold. (B) Biochemical association of HIV Gag sectors inferred using alternative co-evolution methods available in the literature (reviewed in [13]). Specifically, the inferred sectors were based on: 1) Direct coupling analysis (DCA) [67], 2) a mutual information (MI) based method [68], 3) McLachlan based substitution correlation (McBASC) method [69], 4) observed minus expected square (OMES) method [70], and 5) evolutionary trace (ET) method [71]. The MI and DCA methods were implemented using the code provided in [67]. For a fair comparison, the similarity-based sequence weighting of DCA was not applied; however, a pseudo-count value of 0.5 was used (as specified in [67]) to avoid singularity issues during inversion of the covariance matrix in DCA. The McBASC [69] and OMES [70] methods were implemented using the code provided in [72]. The ET method was run from the web-based server provided at http://lichtargelab.org/software/ETserver. None of these methods, except the ET method, were originally designed to explicitly produce sectors of co-evolving residues, but to simply assign a score to each pair of residues in the protein, with a high score indicating a high probability of the associated pair to be in contact in the tertiary structure. Nonetheless, we formed a single sector based on these pairwise scores. This was done by aggregating those pairs of residues deemed to be significantly interacting, corresponding to pairs with an associ [file pcbi.1006409.s008.pdf]

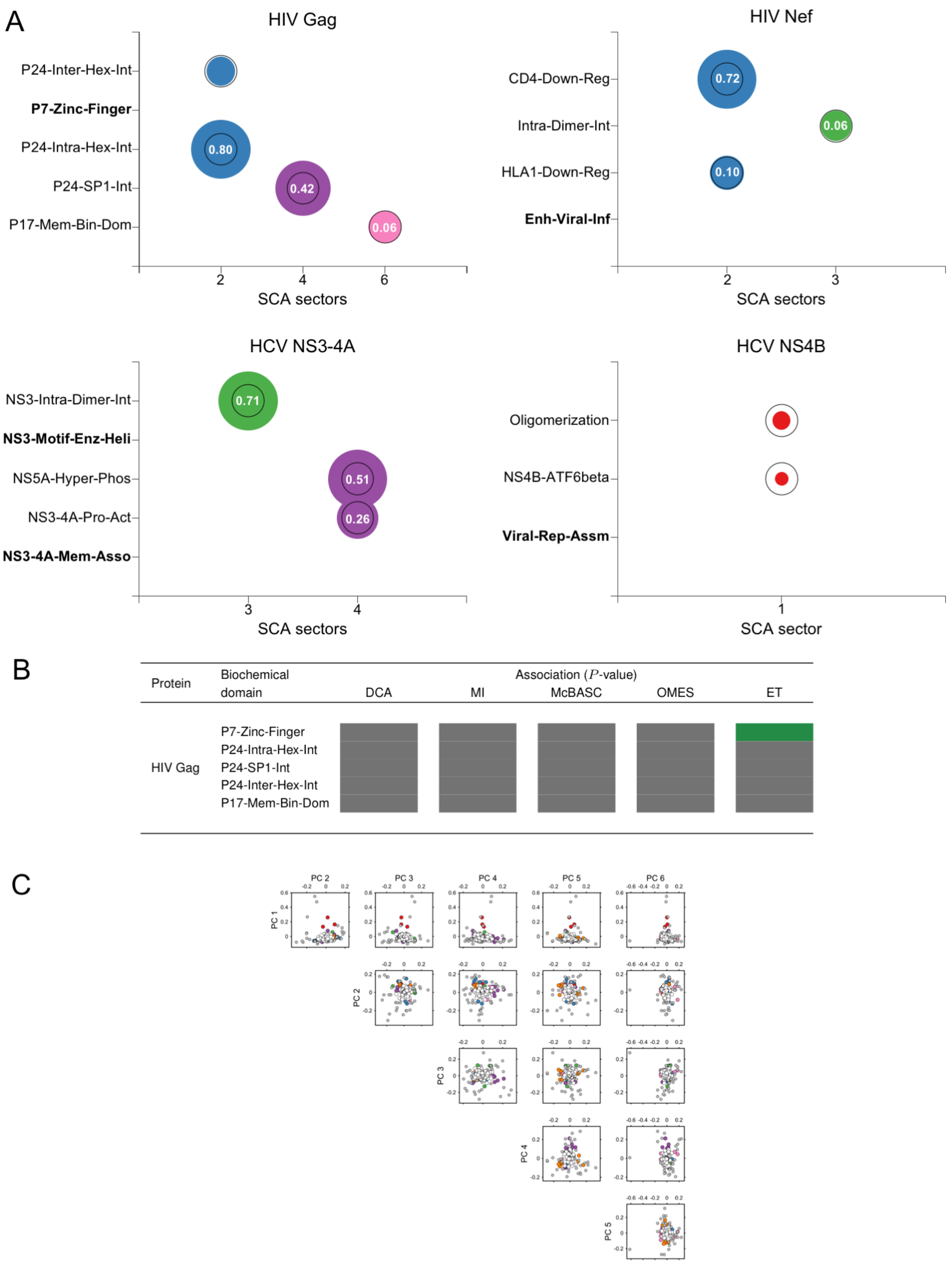
A
HIV Gag
P24-Inter-Hex-Int
P7-Zinc-Finger
P24-Intra-Hex-Int
P24-SP1-Int
P17-Mem-Bin-Dom
0.80
0.42
0.06
2
4
6
SCA sectors
HIV Nef
CD4-Down-Reg
Intra-Dimer-Int
HLA1-Down-Reg
Enh-Viral-Inf
0.72
0.06
0.10
2
3
SCA sectors
HCV NS3-4A
NS3-Intra-Dimer-Int
NS3-Motif-Enz-Heli
NS5A-Hyper-Phos
NS3-4A-Pro-Act
NS3-4A-Mem-Asso
0.71
0.51
0.26
3
4
SCA sectors
HCV NS4B
Oligomerization
NS4B-ATF6beta
Viral-Rep-Assm
1
SCA sector
B
Protein
Biochemical domain
Association (P-value)
DCA
MI
McBASC
OMES
ET
HIV Gag
P7-Zinc-Finger
P24-Intra-Hex-Int
P24-SP1-Int
P24-Inter-Hex-Int
P17-Mem-Bin-Dom
C
PC 1
PC 2
PC 3
PC 4
PC 5
PC 6

Fig S5 ***(preceding page)*****. Comparison with other co-evolutionary methods.** (A) Individual associations of sectors produced by the SCA method proposed in [1] with the biochemical domains of the studied viral proteins; compare with Figs 3 and 4C. The sectors are colored according to the scheme in Fig 2A. Only the sectors having statistically significant association with any biochemical domain are presented. The P-values associated with non-significant associations ($P > 0.05$) are displayed inside the black circle while the biochemical domains having no association with any inferred sector ($P = 1$) are shown in bold. (B) Biochemical association of HIV Gag sectors inferred using alternative co-evolution methods available in the literature (reviewed in [2]). Specifically, the inferred sectors were based on: 1) Direct coupling analysis (DCA) [3], 2) a mutual information (MI) based method [4], 3) McLachlan based substitution correlation (McBASC) method [5], 4) observed minus expected square (OMES) method [6], and 5) evolutionary trace (ET) method [7]. The MI and DCA methods were implemented using the code provided in [3]. For a fair comparison, the similarity-based sequence weighting of DCA was not applied; however, a pseudo-count value of 0.5 was used (as specified in [3]) to avoid singularity issues during inversion of the covariance matrix in DCA. The McBASC [5] and OMES [6] methods were implemented using the code provided in [8]. The ET method was run from the web-based server provided at `http://mammoth.bcm.tmc.edu/ETserver.html`. None of these methods, except the ET method, were originally designed to explicitly produce *sectors* of co-evolving residues, but to simply assign a score to each pair of residues in the protein, with a high score indicating a high probability of the associated pair to be in contact in the tertiary structure. Nonetheless, we formed a single sector based on these pairwise scores. This was done by aggregating those pairs of residues deemed to be *significantly* interacting, corresponding to pairs with an associated score larger than $\beta = 2$ standard deviations above the mean of the overall distribution of scores. Different choices of β yielded qualitatively similar results (not shown). The ET method combines information of the cross-sectional conservation (single-residue conservation in the MSA) and the conserved residues in different branches of the phylogenetic tree (associated with the input MSA) to assign a score to each protein residue. In this algorithm, a lower score reflects higher importance of the residue. Thus, we formed a sector by including those 20% of residues with the lowest scores (as mentioned in [7]). The sector predicted by these methods, except the ET method, showed no statistically significant association to any biochemical domain in HIV Gag. The sector predicted by the ET method was found to be associated with the P7-Zinc-Finger domain. Note that we also tested the multiple correspondence analysis (MCA) based S3det co-evolution method [9] using the web-based server provided at `http://treedetv2.bioinfo.cnio.es/treedet/index.html`. However, no results could be obtained due to its high computational complexity when applied to the (large) Gag protein. (B) Biplots of all possible pairs of the top six PCs—after discarding the leading eigenvector representing the phylogenetic effect—of the SCA matrix, used to form HIV Gag sectors with SCA [1]. Sector residues, overlapping residues, and non-sector residues are represented with the same color scheme of Fig 2A.

References

1. Halabi N, Rivoire O, Leibler S, Ranganathan R. Protein sectors: Evolutionary units of three-dimensional structure. Cell. 2009;138(4):774–786. doi:10.1016/j.cell.2009.07.038.

2. de Juan D, Pazos F, Valencia A. Emerging methods in protein co-evolution. Nat Rev Genet. 2013;14(4):249–261. doi:10.1038/nrg3414.

3. Morcos F, Pagnani A, Lunt B, Bertolino A, Marks DS, Sander C, et al. Direct-coupling analysis of residue coevolution captures native contacts across many protein families. Proc Natl Acad Sci. 2011;108(49):E1293–E1301. doi:10.1073/pnas.1111471108.

4. Dunn SD, Wahl LM, Gloor GB. Mutual information without the influence of phylogeny or entropy dramatically improves residue contact prediction. Bioinformatics. 2008;24(3):333–340. doi:10.1093/bioinformatics/btm604.

5. Göbel U, Sander C, Schneider R, Valencia A. Correlated mutations and residue contacts in proteins. Proteins. 1994;18(4):309–317.

6. Kass I, Horovitz A. Mapping pathways of allosteric communication in GroEL by analysis of correlated mutations. Proteins. 2002;48(4):611–617. doi:10.1002/prot.10180.

7. Mihalek I, Reš I, Lichtarge O. A family of evolution-entropy hybrid methods for ranking protein residues by importance. J Mol Biol. 2004;336(5):1265–1282. doi:10.1016/j.jmb.2003.12.078.

8. Fodor AA, Aldrich RW. Influence of conservation on calculations of amino acid covariance in multiple sequence alignments. Proteins. 2004;56(2):211–221. doi:10.1002/prot.20098.

9. Rausell A, Juan D, Pazos F, Valencia A. Protein interactions and ligand binding: From protein subfamilies to functional specificity. Proc Natl Acad Sci. 2010;107(5):1995–2000. doi:10.1073/pnas.0908044107.
