# Supplementary material for: Co-evolution networks of HIV/HCV are modular with direct association to structure and function
Source: PLoS Comput Biol. 2018 Sep 7;14(9):e1006409. doi: 10.1371/journal.pcbi.1006409 (PMC6145588; doi:10.1371/journal.pcbi.1006409)
Supplement: S6 Fig — These sectors were inferred in [11] using the HIV Gag sequence data available at the time of publication. For all structural interfaces, the contact-defining distance between the alpha-carbon atoms was set to d = 7Å. The inference method in [11] is similar to the PCA approach [12] tested in this paper, mainly differing in the procedure to form sectors from eigenvectors; specifically, the method in [11] formed sectors from visual inspection of eigenvector biplots, whereas an automated procedure was applied in [12]. Similar to [12], the method in [11] produced relatively large sectors that did not show the highly resolved and modular biochemical association revealed by RoCA (Fig 4). Importantly, no sector reported in [11] was found to be associated with RP. This was due to the fact that the residues in the biochemical domain important in this case (p24-SP1 interface; see Results) were mixed up with the epitope residues associated with LTNP in sector 3. (PDF) [file pcbi.1006409.s009.pdf]

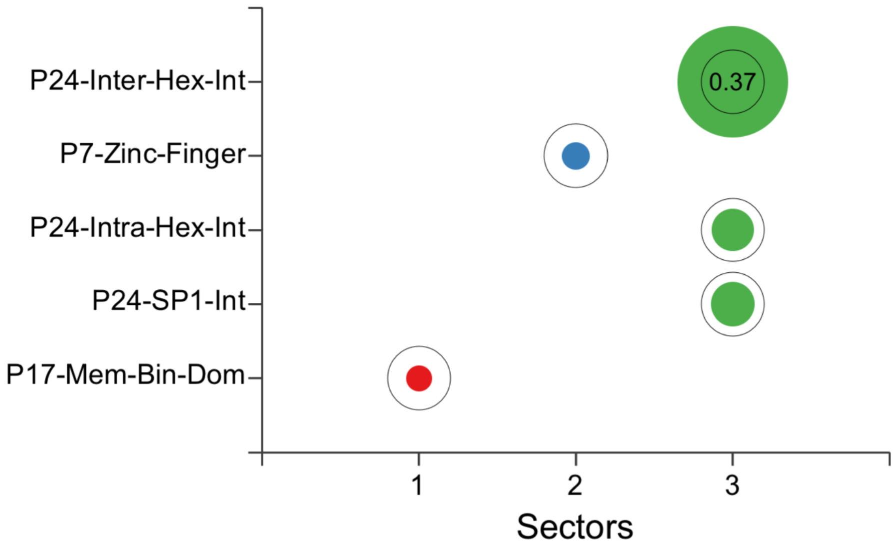

Fig S6. Biochemical association of the previously identified HIV Gag sectors listed in the Supporting Information of [1]. These sectors were inferred in [1] using the HIV Gag sequence data available at the time of publication. For all structural interfaces, the contact-defining distance between the alpha-carbon atoms was set to $d = 7$Å. The inference method in [1] is similar to the PCA approach [2] tested in this paper, mainly differing in the procedure to form sectors from eigenvectors; specifically, the method in [1] formed sectors from visual inspection of eigenvector biplots, whereas an automated procedure was applied in [2]. Similar to [2], the method in [1] produced relatively large sectors that did not show the highly resolved and modular biochemical association revealed by RoCA (Fig 3). Importantly, no sector reported in [1] was found to be associated with RP. This was due to the fact that the residues in the biochemical domain important in this case (p24-SP1 interface; see *Results*) were mixed up with the epitope residues associated with LTNP in sector 3.

References

1. Dahirel V, Shekhar K, Pereyra F, Miura T, Artyomov M, Talsania S, et al. Coordinate linkage of HIV evolution reveals regions of immunological vulnerability. Proc Natl Acad Sci. 2011;108(28):11530–11535. doi:10.1073/pnas.1105315108.

2. Quadeer AA, Louie RHY, Shekhar K, Chakraborty AK, Hsing IM, McKay MR. Statistical linkage analysis of substitutions in patient-derived sequences of genotype 1a hepatitis C virus nonstructural protein 3 exposes targets for immunogen design. J Virol. 2014;88(13):7628–44. doi:10.1128/JVI.03812-13.
