# Supplementary material for: Co-evolution networks of HIV/HCV are modular with direct association to structure and function
Source: PLoS Comput Biol. 2018 Sep 7;14(9):e1006409. doi: 10.1371/journal.pcbi.1006409 (PMC6145588; doi:10.1371/journal.pcbi.1006409)
Supplement: S7 Fig — Circular plots showing both the short and long-range mutational interactions in the six RoCA sectors predicted for HIV Gag. The interactions among residues in a sector are represented with colored lines following the scheme specified in Fig 3A. For better visualization, only the strong interactions (|C^ij|>0.1) involved in each sector are shown. (PDF) [file pcbi.1006409.s010.pdf]

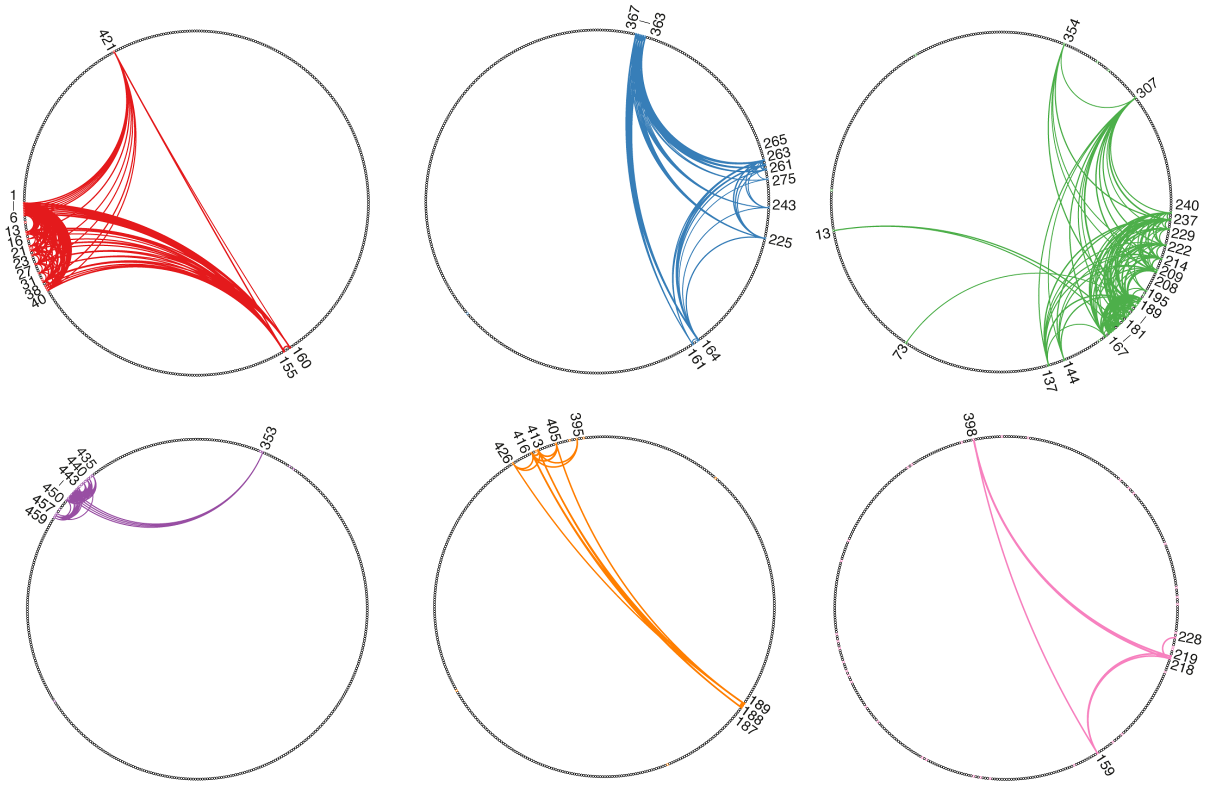


**Fig S7. Presence of long-range interactions in the predicted RoCA sectors.** Circular plots showing both the short and long-range mutational interactions in the six RoCA sectors predicted for HIV Gag. The interactions among residues in a sector are represented with colored lines following the scheme specified in Fig 2A. For better visualization, only the strong interactions ($|\hat{C}_{ij}| > 0.1$) involved in each sector are shown.
