# Supplementary material for: Co-evolution networks of HIV/HCV are modular with direct association to structure and function
Source: PLoS Comput Biol. 2018 Sep 7;14(9):e1006409. doi: 10.1371/journal.pcbi.1006409 (PMC6145588; doi:10.1371/journal.pcbi.1006409)
Supplement: S8 Fig — A high positive Pearson correlation r (close to 1) with very high statistical significance (very small P) is obtained between the entropy per residue of the amino acid MSA (Hi) and that of the binarized MSA (Hibin) for (A) HIV Gag, (B) HIV Nef, (C) HCV NS3-4A, and (D) HCV NS4B. This demonstrates that the binary MSA is a good approximation of the amino acid MSA for the considered viral proteins. For details on the entropy computation, see [12]. (PDF) [file pcbi.1006409.s011.pdf]

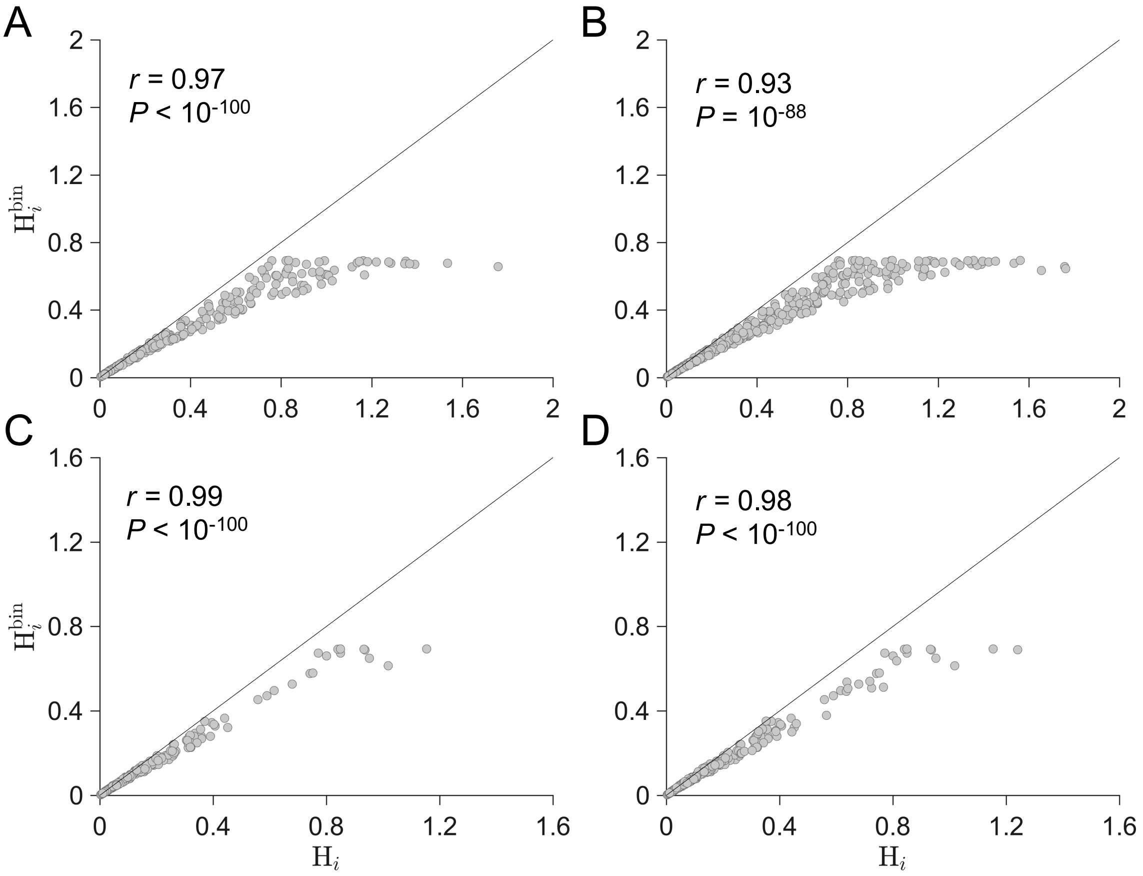

Fig S8. Comparison of the entropy per residue of the amino acid MSA and that of the binarized MSA for (A) HIV Gag, (B) HIV Nef, (C) HCV NS3-4A, and (D) HCV NS4B. A high positive Pearson correlation r (close to 1) with very high statistical significance (very small P) is obtained between the entropy per residue of the amino acid MSA (H_i) and that of the binarized MSA ($\mathrm{H}_i^{\mathrm{bin}}$) in all cases; for details on the entropy computation, see [1]. This demonstrates that the binary MSA is a good approximation of the amino acid MSA for the considered viral proteins.

References

1. Quadeer AA, Louie RHY, Shekhar K, Chakraborty AK, Hsing IM, McKay MR. Statistical linkage analysis of substitutions in patient-derived sequences of genotype 1a hepatitis C virus nonstructural protein 3 exposes targets for immunogen design. J Virol. 2014;88(13):7628–44. doi:10.1128/JVI.03812-13.
