# Supplementary material for: Co-evolution networks of HIV/HCV are modular with direct association to structure and function
Source: PLoS Comput Biol. 2018 Sep 7;14(9):e1006409. doi: 10.1371/journal.pcbi.1006409 (PMC6145588; doi:10.1371/journal.pcbi.1006409)
Supplement: S9 Fig — The RoCA sectors were inferred from the same sequence data used by [14]. The relevant biochemical domains in this case were identified as distinct sets of residues involved with thermal stability [74, 75], basic chemical function of this enzyme family, and catalytic specificity [76, 77]. An important distinction here with respect to the HIV and HCV proteins is that the leading eigenvector obtained in the estimation of the SCA and RoCA sectors was associated with two biochemical functions; thermal stability and enzymic activity. In [14], two sub-sectors were formed from this eigenvector based on the sign of its elements; specifically, the red sub-sector was formed using the positive elements, while the blue sub-sector was formed using the negative elements. The same strategy was applied for RoCA, resulting in the corresponding sector 1 being divided into two sub-sectors, 1a and 1b. (PDF) [file pcbi.1006409.s012.pdf]

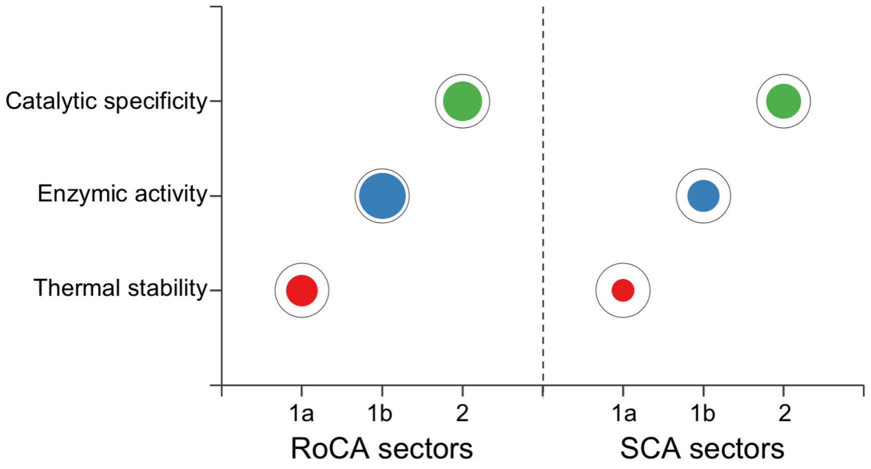

Fig S9. Biochemical association of the RoCA sectors obtained for the S1A serine protease family, in comparison with the SCA sectors produced by [1]. The RoCA sectors were inferred from the same sequence data used by [1]. The relevant biochemical domains in this case were identified as distinct sets of residues involved with thermal stability [2, 3], basic chemical function of this enzyme family, and catalytic specificity [4, 5]. An important distinction here with respect to the HIV and HCV proteins is that the leading eigenvector obtained in the estimation of the SCA and RoCA sectors was associated with two biochemical functions; thermal stability and enzymic activity. In [1], two sub-sectors were formed from this eigenvector based on the sign of its elements; specifically, the red sub-sector was formed using the positive elements, while the blue sub-sector was formed using the negative elements. The same strategy was applied for RoCA, resulting in the corresponding sector 1 being divided into two sub-sectors, 1a and 1b.

References

1. Halabi N, Rivoire O, Leibler S, Ranganathan R. Protein sectors: Evolutionary units of three-dimensional structure. Cell. 2009;138(4):774–786. doi:10.1016/j.cell.2009.07.038.

2. Hedstrom L, Perona JJ, Rutter WJ. Converting trypsin to chymotrypsin: Residue 172 is a substrate specificity determinant. Biochemistry. 1994;33(29):8757–8763. doi:10.1021/bi00195a017.

3. Hedstrom L. Serine protease mechanism and specificity. Chem Rev. 2002;102(12):4501–4524. doi:10.1021/cr000033x.

4. Huntington JA, Esmon CT. The molecular basis of thrombin allostery revealed by a 1.8Å structure of the "slow" form. Structure. 2003;11(4):469–479. doi:10.1016/S0969-2126(03)00049-2.

5. Guinto ER, Caccia S, Rose T, Fütterer K, Waksman G, Di Cera E. Unexpected crucial role of residue 225 in serine proteases. Proc Natl Acad Sci. 1999;96(5):1852–1857. doi:10.1073/pnas.96.5.1852.
