# Supplementary material for: Co-evolution networks of HIV/HCV are modular with direct association to structure and function
Source: PLoS Comput Biol. 2018 Sep 7;14(9):e1006409. doi: 10.1371/journal.pcbi.1006409 (PMC6145588; doi:10.1371/journal.pcbi.1006409)
Supplement: S10 Fig — This result shows that the residues within each studied viral protein are well-mixed with respect to conservation and thus, high conservation does not generally imply biochemical importance. (PDF) [file pcbi.1006409.s013.pdf]

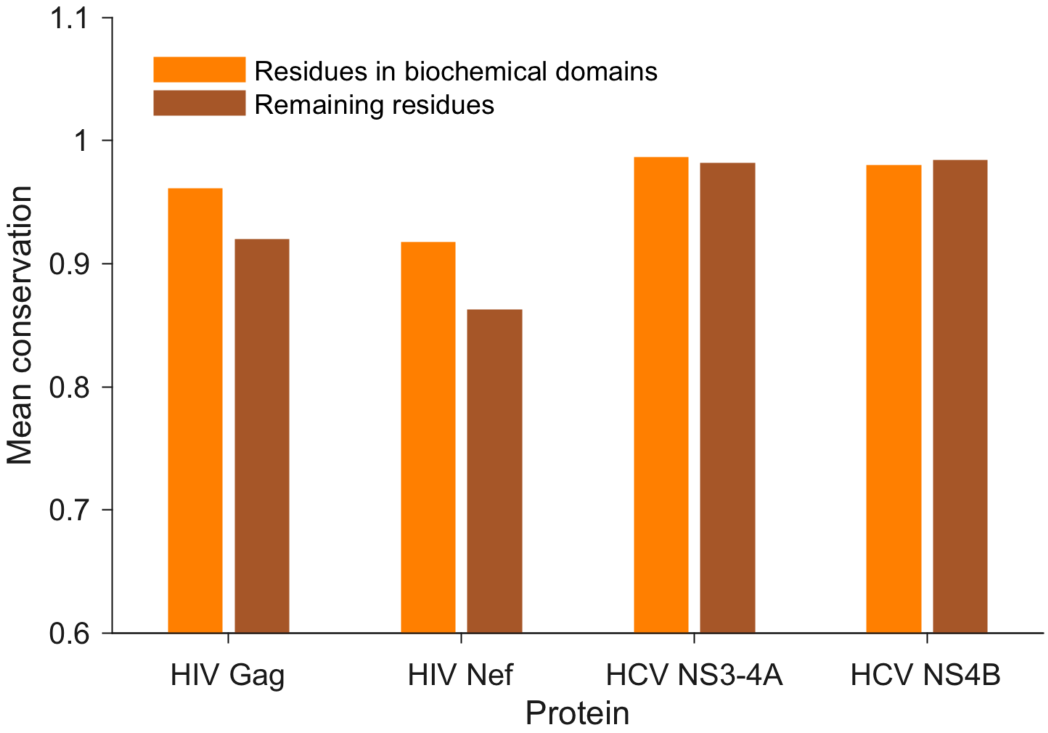

Fig S10. Comparison of the mean conservation of residues within the biochemical domains and those that do not belong to any biochemical domain in each studied viral protein. This result shows that the residues within each studied viral protein are well-mixed with respect to conservation and thus, high conservation does not generally imply biochemical importance.
