# Supplementary material for: Co-evolution networks of HIV/HCV are modular with direct association to structure and function
Source: PLoS Comput Biol. 2018 Sep 7;14(9):e1006409. doi: 10.1371/journal.pcbi.1006409 (PMC6145588; doi:10.1371/journal.pcbi.1006409)
Supplement: S11 Fig — The parameters used in the simulation study (see S2 Text for details) were M = 500 residues and r = 5 non-overlapping units Si of size 12%, 10%, 8%, 6%, and 4% of M, respectively for i = 1, …, 5. The corresponding ℓi were set to equally spaced values between ℓ1 = 6 and ℓ5 = 4, and to model the phylogenetic effect, we set ℓ0 = 8. The ratio of the number of samples to the number of residues was fixed at NM = 4 and the simulation was run for 500 Monte Carlo realizations. (A) The maximum percentage mismatch PMmax between sectors formed using the PCs estimated at a particular Corr-ITSPCA iteration and the corresponding units. PMmax decreases as the number of iterations increases, demonstrating that the iterative procedure in Corr-ITSPCA helps to accurately predict the true units. Here, the intermediate iteration corresponds to half of the total number of iterations Corr-ITSPCA took to converge in each Monte Carlo realization. (B-D) Illustration of the convergence of RoCA sectors to the corresponding units in a single Monte Carlo realization for the simulation setting of (A). Snapshot of (B) PCs representing the true units (here, the first PC represents phylogeny while the subsequent five PCs represent the five units), (C) PCs of the sample correlation matrix (constructed using the phylogeny-filtered MSA) used in forming PCA sectors, and (D) PCs at first, fourth, and eight (last) iteration of the Corr-ITSPCA method. (PDF) [file pcbi.1006409.s014.pdf]

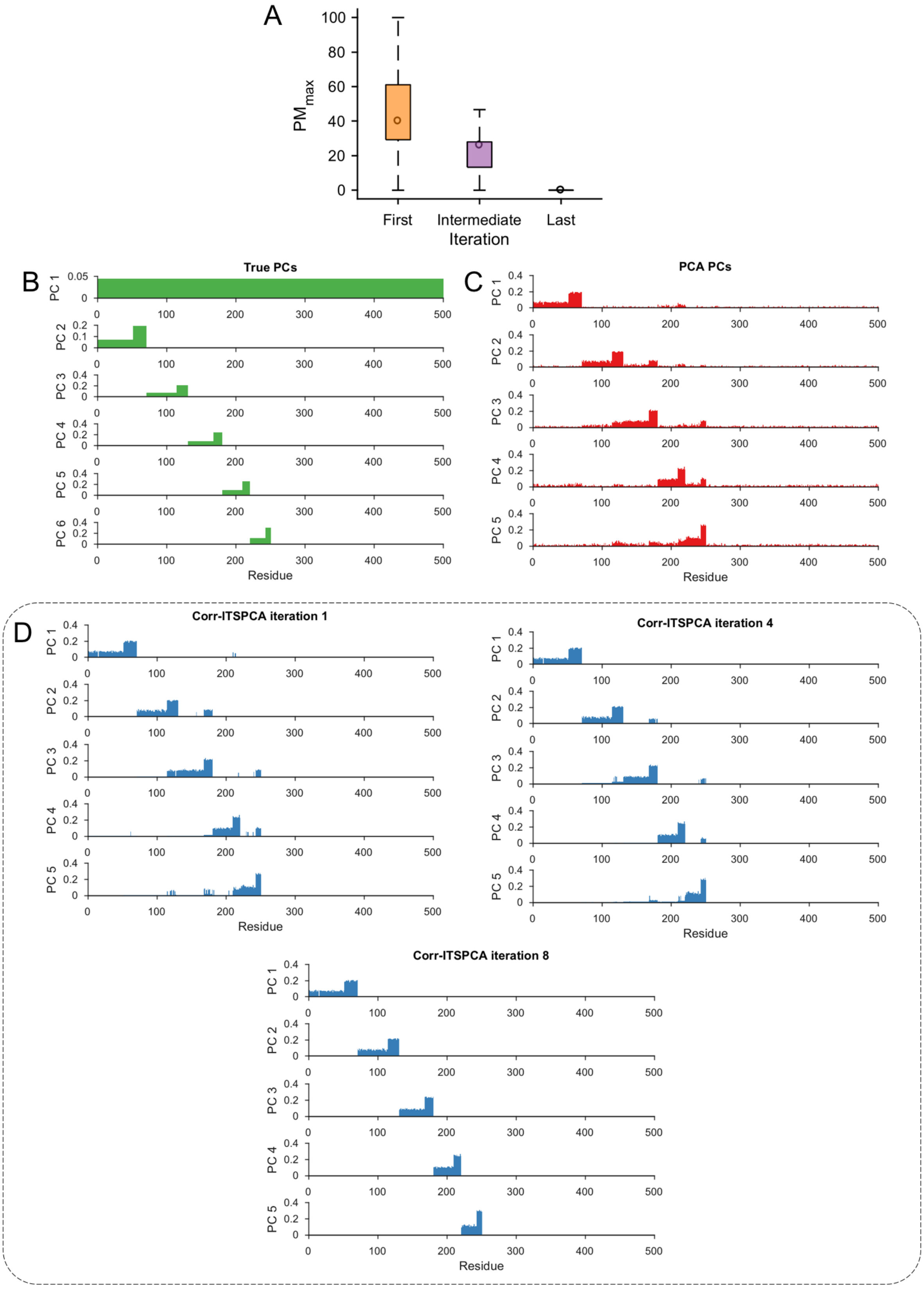
A
B
C
D
PM$_{max}$
First
Intermediate
Iteration
Last
True PCs
PCA PCs
Corr-ITSPCA iteration 1
Corr-ITSPCA iteration 4
Corr-ITSPCA iteration 8
Residue

Fig S11 ***(preceding page)*****. Effect of Corr-ITSPCA iterations on the RoCA sector inference using binary synthetic data.** The parameters used in the simulation study (see S2 Text for details) were $M = 500$ residues and $r = 5$ non-overlapping units S_i of size 12%, 10%, 8%, 6%, and 4% of M, respectively for $i = 1, \ldots, 5$. The corresponding ℓ_i were set to equally spaced values between $\ell_1 = 6$ and $\ell_5 = 4$, and to model the phylogenetic effect, we set $\ell_0 = 8$. The ratio of the number of samples to the number of residues was fixed at $\frac{N}{M} = 4$ and the simulation was run for 500 Monte Carlo realizations. (A) The maximum percentage mismatch $\mathrm{PM}_{\mathrm{max}}$ between sectors formed using the PCs estimated at a particular Corr-ITSPCA iteration and the corresponding units. $\mathrm{PM}_{\mathrm{max}}$ decreases as the number of iterations increases, demonstrating that the iterative procedure in Corr-ITSPCA helps to accurately predict the true units. Here, the intermediate iteration corresponds to half of the total number of iterations Corr-ITSPCA took to converge in each Monte Carlo realization. (B-D) Illustration of the convergence of RoCA sectors to the corresponding units in a single Monte Carlo realization for the simulation setting of (A). Snapshot of (B) PCs representing the true units (here, the first PC represents phylogeny while the subsequent five PCs represent the five units), (C) PCs of the sample correlation matrix (constructed using the phylogeny-filtered MSA) used in forming PCA sectors, and (D) PCs at first, fourth, and eight (last) iteration of the Corr-ITSPCA method.
