# Supplementary material for: Co-evolution networks of HIV/HCV are modular with direct association to structure and function
Source: PLoS Comput Biol. 2018 Sep 7;14(9):e1006409. doi: 10.1371/journal.pcbi.1006409 (PMC6145588; doi:10.1371/journal.pcbi.1006409)
Supplement: S1 Table — (PDF) [file pcbi.1006409.s015.pdf]

Table S1. List of HLA class I restricted epitopes associated with long-term non-progressors (LTNP) and rapid progressors (RP) in HIV Gag.

No.	Association	Epitope	Restricting HLA	Peptide sequence
1		19–27	B*2705	IRLRPGGKK
2		145–155	A*2501	QAISPRTLNAW
3		147–155	B*5701	ISPRTLNAW
4		162–172	B*5701, B*5703	KAFSPEVIPMF
5		180–188	Cw*0802	TPQDLNTML
6	LTNP	203–212	A*2501	ETINEEAAEW
7		240–249	B*5701, B*5801	TSTLQEQIGW
8		263–272	B*2705	KRWIILGLNK
9		298–306	B*1402	DRFYKTLRA
10		305–313	Cw*0802	RAEQASQEV
1		36–44	B*3501	WASRELERF
2		78–86	A*2902	LYNTVATLY
3	RP	254–262	B*3501	PPIPVGDIY
4		260–267	B*0801	EIYKRWII
5		355–363	B*0702	GPGHKARVL
